# Balancing the risk of major bleeding against vascular disease risk in people without atherosclerotic disease

**DOI:** 10.1136/heartjnl-2024-324841

**Published:** 2025-02-26

**Authors:** Imen Hammami, Marion Mafham, Jonathan Emberson, Alison Offer, Jemma C Hopewell, Jane Armitage, Colin Baigent, Sarah Parish

**Affiliations:** 1 Clinical Trial Service Unit and Epidemiological Studies Unit, University of Oxford Nuffield Department of Population Health, Oxford, UK

**Keywords:** Cardiovascular Diseases, Risk Factors, Risk Assessment

## Abstract

**Background and aims:**

In the primary prevention setting, low-dose aspirin reduces major vascular events (MVEs) by approximately 11% but increases major bleeding (MB) by 40–50%, implying that net benefit will be most evident when the MVE-to-MB ratio is >4. This study aimed to derive cross-validated risk scores for MB and MVE and use the MVE-to-MB ratio to identify groups who may derive differing net benefits from treatment.

**Methods:**

431 167 UK Biobank participants without known atherosclerotic cardiovascular disease at baseline were followed through record linkage for incident MVEs (myocardial infarction, non-haemorrhagic stroke, transient ischaemic attack, arterial revascularisation or vascular death) and MB (gastrointestinal and intracranial bleeds with hospital admission for ≥2 days). Risk scores were derived for MVE and MB using Cox proportional hazards models with cross-validation. Ratios of observed MVE-to-MB rates were calculated across risk categories.

**Results:**

During a median follow-up of 12 years, 18 310 participants suffered an MVE and 5352 an MB. MB risk was highest among participants with frailty, prior bleeds, cancer, liver disease or renal dysfunction, with a 4.3-fold difference in risk between the highest and lowest fifths of MB risk (HR 4.3, 95% CI 3.87 to 4.77). The MVE-to-MB ratio was ≤2.6 in the highest MB risk groups and ≥4 in lower MB risk categories.

**Conclusions:**

The derived models using routinely available disease history and laboratory measurements improved distinction of the MVE-to-MB ratio compared with using conventional models for MB risk including vascular risk factors. Such models can help identify those with moderate MVE risk but low MB risk who may benefit from low-dose aspirin.

**WHAT IS ALREADY KNOWN ON THIS TOPIC**

- Aspirin reduces the risk of major vascular events (MVE) but increases the risk of major bleeding (MB), with uncertainty about its net benefit in primary prevention.
- Prediction of the risk of MB and MVEs could help identify subgroups of individuals with a net benefit from aspirin therapy.

**WHAT THIS STUDY ADDS**

- Risk scores for MB and MVEs were derived from clinical and laboratory data, which enabled partial separation of groups with varying ratios of risk of MVE to MB.
- A ratio of MVE to MB rates below 4 was observed in individuals with the highest bleeding risk, suggesting potential net harm from aspirin use.

**HOW THIS STUDY MIGHT AFFECT RESEARCH, PRACTICE OR POLICY**

- The study supports risk-based decision-making for aspirin use based on predicted bleeding and vascular risks.
- Risk scores derived from routinely collected clinical data could aid in optimising primary prevention strategies.

## Introduction

 Low-dose aspirin for cardiovascular prevention is of net benefit for patients with prior cardiovascular disease (secondary prevention), but among people without known cardiovascular diseases (primary prevention), the net effect remains uncertain. In primary prevention trials, aspirin produces an 11% proportional reduction in major vascular events (MVEs) and a 40–50% proportional increase in major bleeds (MBs).^1^,^3^ Recent guidelines recommend aspirin in people with established atherosclerotic cardiovascular disease (ASCVD), but for others at elevated cardiovascular risk, some guidelines recommend against aspirin use,^4^ while others recommend against initiation in older adults but evaluating the balance of benefit and harm in younger adults or in type 2 diabetes.^5^,^7^ Vascular risk factors are also associated with higher risks of serious bleeds in aspirin primary prevention trials, but the recording of other risk factors in such studies has been limited.^1 2^ Linkage to electronic healthcare data in large biobanks can now provide much more extensive disease history.

The present study uses the UK Biobank prospective study of 0.5 million participants linked to electronic hospital admission data to investigate the risk of bleeding events in relation to the risk of MVEs, history of disease and bleeding, vascular disease risk, measures of frailty and other available biomarkers. It aims to derive cross-validated risk scores for MB and MVEs and use the ratio of the observed MVE-to-MB rates to identify subgroups who may derive differing net benefits from treatment.

## Methods

### Study design and participants

The study design of UK Biobank has been described in detail elsewhere.^8 9^ Briefly, UK Biobank recruited 0.5 million participants aged 40–69 years from 2006 to 2010 across the UK. At assessment centres, participants provided signed consent, answered questions on sociodemographic, lifestyle, environmental and health-related factors, completed a range of physical measures and provided blood and urine samples. Participants have been linked to electronic records for deaths and hospital admissions with diagnoses and procedures (HADP) data available from about 12 years before recruitment until about 12 years after. This report follows the Enhancing the Quality and Transparency of Health Research reporting guidelines.^10^ The present study is restricted to participants without known ASCVD at baseline.

### Outcomes

Outcomes were defined by relevant International Classification of Diseases-10th Revision (ICD-10) or Office of Population Censuses and Surveys (OPCS) codes in any diagnostic code position in records for hospital admissions during follow-up or as the underlying cause of death. MVE was defined as myocardial infarction, stroke or transient ischaemic attack, arterial revascularisation or vascular death excluding any intracranial haemorrhage (online supplemental data table 1). Definitions of MB in clinical trials vary from those restricted to more severe bleeds^11^ to broader definitions.^2 12^ For these analyses, first MBs following recruitment were defined as intracranial and gastrointestinal (GI) bleeds with duration of hospital spell (period of hospitalisation within one healthcare provider; online supplemental eMethods 1) ≥2 days or in death records (online supplemental data table 2). Intraocular bleeds and transfusion were not adequately captured in the available HADP data and so could not be specified in the definitions (see online supplemental eMethods 1 for further details). Additionally, a broader definition of MB was considered and included intracranial, upper GI, lower GI, respiratory, haematuria (in primary diagnostic code position only) and site unknown bleeds, with duration of spell ≥1 day, or in death records as the underlying cause. Minor bleeds were day admissions or haematuria in a secondary diagnostic code position.

### Exposures

Health information at recruitment was supplemented by information from HADP prior to each year of follow-up. ICD-10 codes were grouped into well-established conditions or ICD-10 section ranges (online supplemental eMethods 1; online supplemental data tables 3 and 4). Codes for cardiovascular conditions were included as potential vascular risk factors, and the remainder as potential MB risk factors. A published ICD-10 hospital admission-based frailty risk score,^13^ modified to exclude codes related to history of cardiovascular conditions or bleeding, was included as a bleed risk factor. The aim was to provide a similar level of detail and annual updating of exposures for the two outcomes.

In total, ~75 HADP-derived vascular factors were considered for MVE risk in addition to established vascular risk factors (attained age, sex, systolic and diastolic blood pressure, use of blood pressure-lowering medication, body mass index, smoking status, diabetes and non-high-density lipoprotein cholesterol). About 500 non-vascular factors were considered for MB risk, including the frailty risk score, 40 ‘standard’ factors that would typically be readily available in clinical practice, 47 additional factors available in UK Biobank and 420 factors derived from HADP (online supplemental data tables 4 and 5). The standard and additional factors were cleaned and imputed before analysis (online supplemental eMethods 1). Attained age and the HADP-derived factors were updated annually during a participant’s follow-up; for other variables, only recruitment values were available and were used in all years. The HADP-derived factors were further categorised as within 2 years prior to the start of the year of follow-up or longer ago (online supplemental eMethods 1).

### Statistical analyses

Analyses were restricted to participants with no intracranial bleed, major extracranial bleed or GI ulcer in the 6 months prior to recruitment (as among these people aspirin use may be considered contraindicated) and to those with complete information on established vascular factors (online supplemental efigure 1). The included participants had no known ASCVD at baseline, and observations were censored at the recording of ASCVD during follow-up.

Vascular risk factors for MVE and non-vascular risk factors for MB were identified by sequentially selecting additional variables into base Cox proportional hazards models (online supplemental eMethods 2). The base model for MVE included established vascular risk factors, and that for MB included the established and additional risk factors for first MVE, anticoagulation and antiplatelet use and the hospital frailty risk score at the start of each year of follow-up. An MVE risk score for the absolute annual risk was formed by fitting a Poisson regression to the established and additionally selected risk factors (with 10-fold cross-validation and assessment of calibration and discrimination; online supplemental eMethods 2). For comparison, MVE risk scores based on (1) established risk factors only and (2) the published SCORE2^14^ score were also made (online supplemental eMethods 2).

For MB, a cross-validated risk score for the HR was calculated, since it is the relative risk of MB at a given level of MVE risk that enables classification of participants with higher bleed risk versus lower bleed risk after taking into account vascular risk. HRs for each type of MB by fifths of their respective scores and by the MB score were estimated by Cox regression with adjustment for attained age, sex, anticoagulation and antiplatelet use and MVE risk score. Calculation of the HRs for MB by fifths of MB score was repeated using the broader MB definition (online supplemental eMethods 2).

Observed incidence rates of first MVE and first MB and the ratio of these rates within MVE risk score group (<0.2%/year, 0.2–0.5%/year, 0.5–1%/year and ≥1%/year) specific fifths of MB risk score were calculated among those with no anticoagulant use and corrected to no antiplatelet use at baseline (assuming the HRs for antiplatelet use were similar to those in a recently reported meta-analysis, as studies have not suggested any modification of the relative effects by subgroups,^2 5^ online supplemental eMethods 3). This was repeated using the broader MB definition and using the QBleed^15^ score (which includes vascular risk factors, online supplemental eMethods 4) instead of the MB score.

All analyses used SAS V.9.4 (SAS Institute, 2013).

### Patient and public involvement

During the design phase, UK Biobank included a public consultation addressing questions of public trust.

## Results

### MVE risk factors

Among the 431 167 participants without known prior ASCVD at recruitment, 18 310 (0.3%/year) suffered an MVE during follow-up (online supplemental eFigure 1, table 1), including 672 fatal events. The selection algorithm, after forced inclusion of established vascular risk factors (including age and sex), identified the following additional predictors for MVE: chest pain, other cerebrovascular disease (including unspecified), cardiac valve disease, venous thromboembolism, diabetic complications, hypertension and various cardiovascular diagnostic or therapeutic procedures (excluding revascularisation procedures) (online supplemental data table 6). At recruitment, 40.5% had a predicted risk of MVE <0.2%/year, while 5% had a predicted risk ≥1%/year (online supplemental eTable 1). The MVE risk score demonstrated strong calibration and good discrimination (online supplemental eFigure 2).

**Table 1:** Number of participants with hospital admissions including a bleed or a major vascular event diagnosis among 431 167 participants without known prior ASCVD in UK Biobank

Event type	With events, n	Mean stay, days*	%/year
Major bleed			
Intracranial	2045	17.8	0.033
Gastrointestinal	3360	11.9	0.056
Upper gastrointestinal	2457	13.1	0.041
Lower gastrointestinal	1164	8.7	0.020
Any major bleed	5352	13.4	0.089
Broader definition of major bleed†			
Intracranial	2045	17.8	0.033
Gastrointestinal	4241	9.6	0.072
Upper gastrointestinal	2845	11.4	0.048
Lower gastrointestinal	1703	6.2	0.029
Epistaxis	2131	7.3	0.037
Haematuria	1171	4.1	0.020
Other	59	18.3	0.001
Any major bleed (broader definition)	9266	9.8	0.159
Minor bleeds	28 115	0.7	0.542
Major vascular events			
Myocardial infarction	7598	5.7	0.132
Ischaemic stroke	5275	10.9	0.082
Transient ischaemic attack	2577	2.6	0.043
Revascularisation	6258	3.2	0.111
Any major vascular event	18 310	6.9	0.312

* Mean stay of the spell in which the bleed occurred.

† Including intracranial, gastrointestinal, respiratory, haematuria and not elsewhere classified bleeds with a duration of hospital spell ≥1 day. Fatal major bleeds and major vascular events (with or without hospitalisation) were 452 and 672, respectively.

ASCVD, atherosclerotic cardiovascular disease.

Compared with an MVE risk score based on established risk factors alone or the SCORE2 score, the new score was better at identifying those at highest risk (online supplemental eTable 2).

### Rates of MB

**Figure 1:**
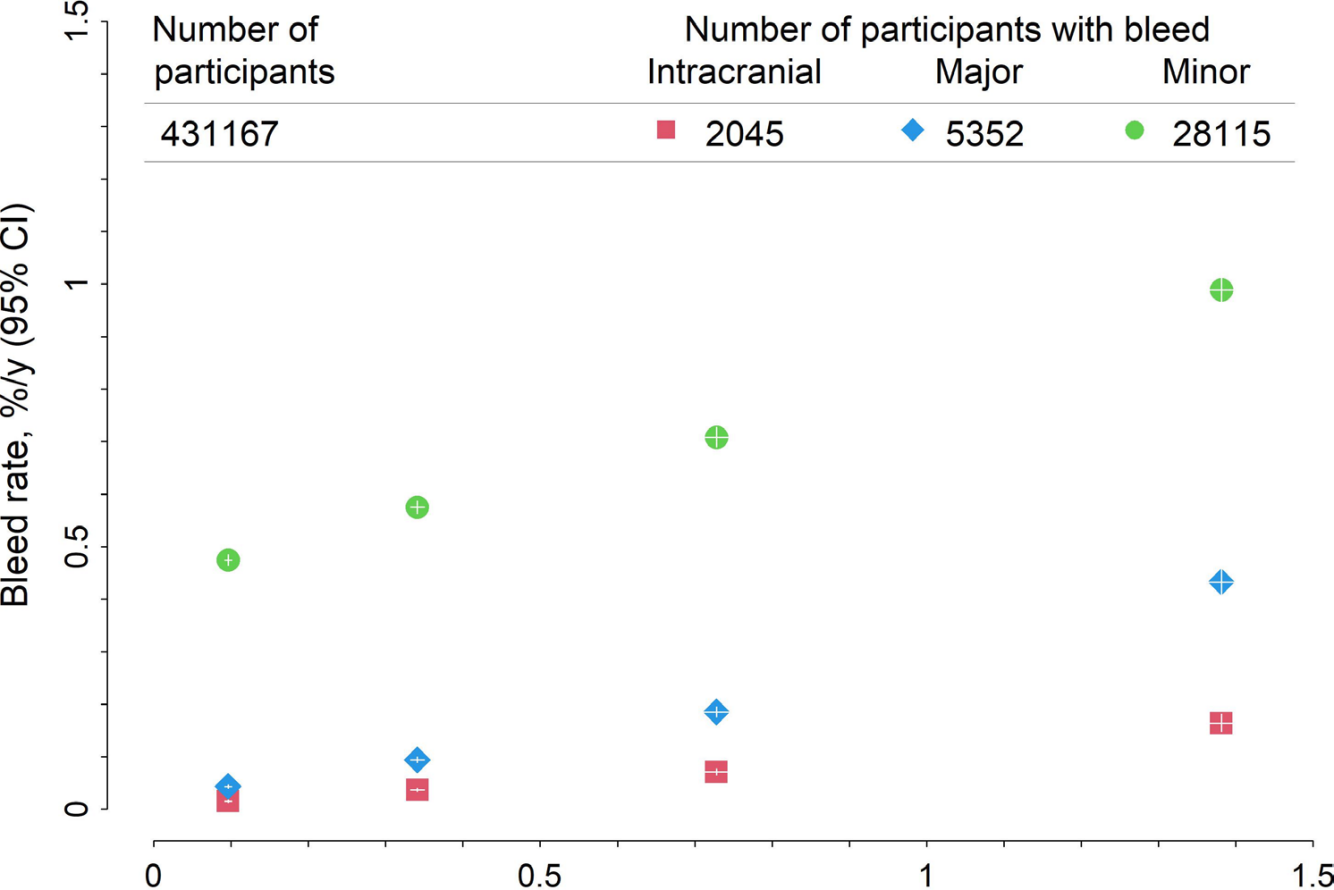
Observed bleed versus major vascular event rates within predicted major vascular event risk groups. Person-years at risk were categorised into four groups of predicted major vascular event risk, <0.2%, ≥0.2%<0.5%, ≥0.5%<1%, and ≥1% per year, based on the major vascular event risk score.

5352 (0.09%/year) participants suffered an MB (including 2045 intracranial, 2457 upper GI and 1164 lower GI) (table 1). A fatal bleed occurred in 452 participants, with the majority being intracranial (387 (86%)), and a minor bleed occurred in 28 115 (0.54%/year) participants. Observed rates of major and minor bleed increased linearly with observed rates of MVE (figure 1). By the broader definition, 9266 (0.16%/year) participants suffered an MB (including 2045 intracranial, 2845 upper GI and 1703 lower GI).

### MB risk factors

The selection algorithm, adjusting for vascular risk factors (including age and sex), antiplatelet and anticoagulant use, identified the following non-vascular risk factors for MB: frailty risk score, further measures of frailty, history of bleed, cancer, liver disease, GI disease or investigations, head trauma, kidney and liver function biomarkers and abnormal blood biomarkers (online supplemental data table 7).

**Figure 2:**
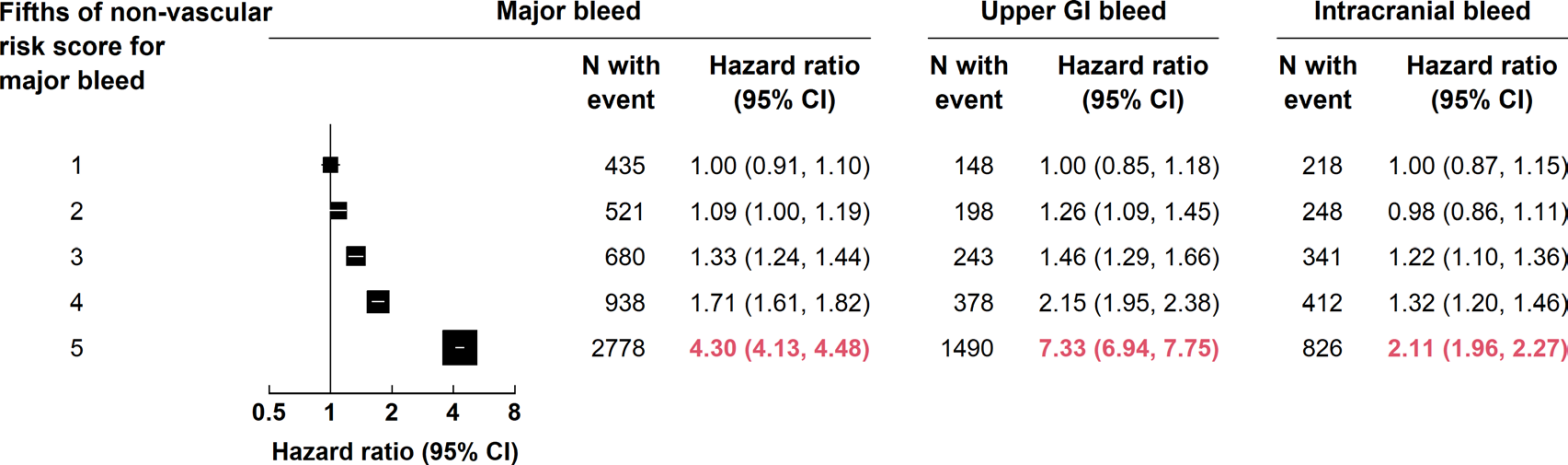
HRs for major, upper gastrointestinal (GI) and intracranial bleed, associated with fifths of non-vascular risk score for major bleed. HRs are adjusted for attained age, sex, anticoagulation and antiplatelet use and major vascular event risk score (both continuous and categorical forms) and plotted as squares, with the size of each square proportional to the amount of statistical information that was available; the horizontal lines represent 95% CIs based on floating absolute risks. The CIs for the top fifth relative to the bottom fifth as a fixed reference group (without floating absolute risks) are 4.30 (3.87, 4.77) for major bleed, 7.33 (6.16, 8.73) for upper GI bleed and 2.11 (1.80, 2.47) for intracranial bleed.

An MB risk score derived from the identified factors distinguished a 4.3-fold difference in MB risk across fifths of the score, with the highest fifth, which included over half the MBs, showing a sharp increase in bleed risk (figure 2). Furthermore, it distinguished a 2.1-fold difference in the risk of intracranial bleed, and 7.3-fold and 5.1-fold differences in the risks of upper and lower GI bleeds, respectively (figure 2, online supplemental eTable 3). However, although the approach included adjustment for the MVE risk score, the MB risk score was also associated with a 1.9-fold difference in risk of MVE across fifths (online supplemental eTable 3).

Most factors selected into the MB risk score were derived from either HADP data or from standard factors routinely available in clinical practice (online supplemental data tables 4 and 5). In a sensitivity analysis, restricting selection to these factors, the derived risk score for MB distinguished a 4.0-fold difference in risk of MB across fifths of the score (online supplemental eFigure 3;online supplemental data table 8).

**Figure 3:**
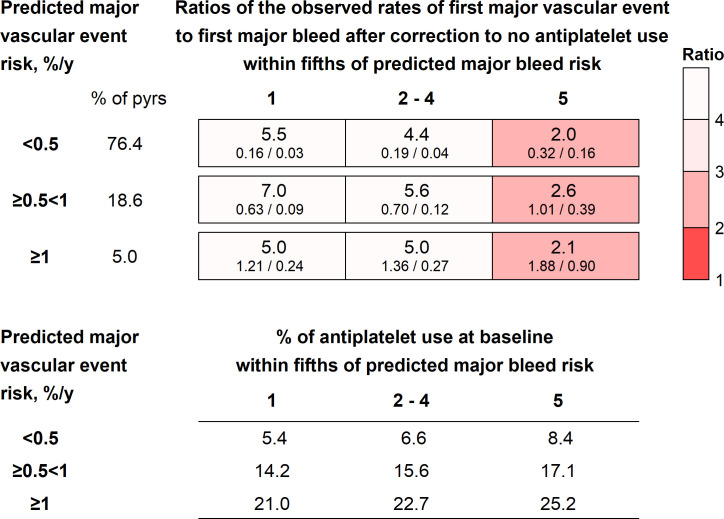
Observed major vascular event and major bleed rates and their ratios by predicted major vascular event and major bleed risks, after correction to no antiplatelet use. Predicted major bleed risk cut-offs were based on major bleed risk score quintiles calculated separately within each major vascular event risk group. Values in bars are the ratio (in large type) and the major vascular event rate (%/year)/major bleed rate (%/year) underneath. pyrs, person-years at risk.

Figure 3 (and online supplemental eTable 4) show, among participants without anticoagulation use, observed MVE and MB rates after correction to no antiplatelet use in a nine-way classification by three categories of predicted MVE risk crossed with three categories of predicted MB risk (fifths within each MVE risk group, with the three middle fifths combined). The ratio of the observed rates of MVE to MB was 2.0 to 2.6 in the groups with the highest predicted bleed risk and 4.4 to 7.0 in the remaining groups (figure 3). Antiplatelet use at recruitment increased with predicted MVE risk (varying from 5.4% to 25.2%) (figure 3). Proton-pump inhibitor (PPI) use increased with MB risk (online supplemental eTable 5).

Using the broader MB definition, MB rates were higher and the ratios of MVE-to-MB rates lower, but among those at predicted MVE risk ≥0.5%/year, in whom aspirin may be considered, the ratios remained ≥3.5 except in the highest fifth of predicted bleed risk where they varied from 1.2 to 1.3 (online supplemental eTable 3 and eFigure 4). Fifths of the QBleed score distinguished a 6.0-fold difference in MB risk but were also strongly associated with MVE risk (4.2-fold difference; online supplemental eFigure 5). In the cross-tabulation of predicted MVE and MB risk using the QBleed score, the ratio of observed MVE-to-MB rates varied little across the bleed risk categories (2.6 to 3.0 among those at highest bleed risk and 2.4 to 5.0 in the remaining groups) (online supplemental eFigure 6).

## Discussion

In this large study with 5352 participants without known prior ASCVD suffering an incident MB, the rates of both MB and minor bleeds increased with MVE risk. Despite this, after adjusting for MVE risk, a risk score for MB, based on non-vascular risk factors (mainly frailty, history of bleeding and cancer), distinguished a 4.3-fold difference in MB risk across fifths and a 7.3-fold difference in the risk of major upper GI bleed. The ratio of the observed rates of MVE to MB varied from 2 to 7 using the main definition. This allowed categories of participant to be distinguished in whom the effects of antiplatelet treatment, which prevents vascular events but causes bleeds, would be expected to have less or more net benefit.

In primary cardiovascular disease prevention, aspirin use is associated with about an 11% reduction in MVE risk compared with 40–50% excess risk of MB, with no detected variation in these proportional effects across subgroups.^1^,^3^ Therefore, in categories of people where the ratio of MVE-to-MB rates is around 4, aspirin may produce a similar absolute reduction in MVEs and excess of MBs, but where the ratio is much <4, it may produce a smaller absolute reduction in MVEs than excess of MBs. The ratio was >4 except among those in the highest fifth of predicted bleed risk, where it was ≤2.6 (figure 3). When the broader definition of MB was used, the ratios of MVE to bleed rates were lower, but among those at predicted MVE risk ≥0.5%/year, in whom aspirin may be considered, the ratios remained >3.5 except among those in the highest fifth of predicted bleed risk. Therefore, these analyses suggest that the likely absolute benefit and harm with aspirin are balanced except in those in the highest bleed risk group, where the hazards may outweigh the benefits.

The absolute rates of these events, and therefore the likely absolute effects of aspirin treatment, are dependent on the definitions of these two types of outcomes. This study aimed to identify hospital admissions for MVE or bleed, using a main definition of MB comparable to Bleeding Academic Research Consortium (BARC) and other bleeding scales.^11 16^ However, in studies using a less stringent definition of MB, such as those used in recent primary prevention trials,^17 18^ the absolute rates of MB would be much higher. Information on transfusion or blood results is not available in HADP data, and evaluation of more severe bleeds was not possible in this study. The only available factors indicating severity were site of bleeding and duration of hospital stay. In a study with more detailed information, intracranial and GI bleeds were more likely to be classified as major or life-threatening than other bleeds.^19^ Therefore, a pragmatic definition was considered, which included hospitalisations involving an intracranial or GI bleed and ≥2 nights in hospital. HADP data may miss up to one-third of MBs,^19^ but these methods may also underestimate MVEs, since events managed as outpatients, such as less severe cerebrovascular events, would not be captured.^20^ A previous report which compared the reliability of MB outcomes in UK routine data (defined similarly to here) and trial adjudicated follow-up in the A Study of Cardiovascular Events in Diabetes (ASCEND) randomised trial found moderate overlap, with both sources identifying a similar number of additional MBs.^21^ Notwithstanding this, MBs ascertained from UK routine data yielded relative and absolute treatment effects similar to adjudicated follow-up, providing support for the validity of using such routine data definitions of MB.

Almost half of MBs in this study were upper GI and could potentially be prevented with PPI treatment, the use of which varied from 4% to 21% among those at the lowest to highest risk of MVE and MB. A meta-analysis of short-term trials (median follow-up of 1.4 months) suggested PPIs may reduce upper GI bleeds by 60% (95% CI 50% to 68%), but may have overestimated the benefits owing to small study bias.^22^ By contrast, the Cardiovascular Outcomes for People Using Anticoagulation Strategies (COMPASS) trial of pantoprazole versus placebo (mean follow-up ~3 years) found a more modest protective effect of PPI therapy (12% (95% CI −15% to 33%) reduction in the primary outcome which included upper GI bleed, obstruction or perforation).^23^ However, the broad definition of upper GI event, exclusion of patients at high bleed risk and wide CI are a limitation of COMPASS. Thus, estimation of the impact of PPI use on upper GI bleed rates is difficult, and further studies are required.

The present study has several limitations. First, the MVE and MB risk scores were derived and applied in the same cohort with internal validation. Validation in another cohort to demonstrate generalisability would be required before implementation in clinical practice. Second, although the UK Biobank cohort includes a broad spectrum of people, it tends to be healthier than the general population, and so absolute rates of MVE and MB may be lower than in the general population. The version of the MB risk score developed in the present study based on measures available from data routinely collected in clinical practice could be easily replicated and validated in available UK healthcare datasets (online supplemental data table 8). Code for forming the scores from defined data can be provided on request.

This study has several strengths. It included a large number of MB events and an extensive range of exposures, particularly of prior disease. In contrast to previous bleed risk scores, such as HAS-BLED^24^ and QBleed,^15^ which include vascular risk factors, the new MB risk score in this study was developed from *non-vascular* risk factors (ie, factors other than established vascular risk factors or diagnoses) after controlling for predicted vascular risk. Despite this, the MB risk score was still somewhat associated with MVE risk, suggesting that it may not be possible to greatly separate these closely correlated hazards. (Thus, the ‘non-vascular’ risk factors and MB were predictors of vascular risk to some degree.) However, we demonstrated that the newly derived score achieved better discrimination of the ratio of MVE-to-MB rates than the QBleed score. The new score used factor information updated annually, where available, to better represent the clinical situation in which a patient’s risk would be estimated from their current risk factor levels and history.

The present findings identify a group of individuals without known prior ASCVD at the highest predicted MB risk, for whom the hazards associated with antiplatelet use are likely to outweigh the benefits. Among most individuals without known ASCVD, however, the absolute benefits and hazards are closely balanced, but a strategy of antiplatelet use with PPI protection among selected individuals without prior cancer or minor bleed may be worthwhile.

## Supplementary material

10.1136/heartjnl-2024-324841 online supplemental file 1

10.1136/heartjnl-2024-324841 online supplemental file 2

## Data Availability

Data may be obtained from a third party and are not publicly available.
