# Supplementary material for: Balancing the risk of major bleeding against vascular disease risk in people without atherosclerotic disease
Source: Heart. 2025 Feb 26;111(15):e324841. doi: 10.1136/heartjnl-2024-324841 (PMC12322474; doi:10.1136/heartjnl-2024-324841)
Supplement: online supplemental file 1 [file heartjnl-111-15-s001.pdf]

Supplementary online content

eMethods 1. Data preparation

eMethods 2. Identifying vascular predictors of major vascular event and non-vascular predictors of major bleed

eMethods 3. Correction to no antiplatelet use

eMethods 4. Implementation of the QBleed score

eTable 1. Observed major vascular event rates by predicted major vascular event risk score.

eTable 2. Observed major vascular event rates by predicted major vascular event risk scores derived from established and additional vascular factors and SCORE2.

eTable 3. Hazard ratios for lower gastrointestinal (GI) bleed, broader major bleed, and major vascular events, respectively, associated with fifths of non-vascular risk score for major bleed.

eTable 4. Observed major vascular event and major bleed rates by predicted major vascular event and major bleed risks after correction to no antiplatelet use.

eTable 5. Proportion of proton pump inhibitors use (%) at baseline by predicted major vascular event and major bleed risks.

eFigure 1. Flow diagram for participants included and the various reasons for exclusion.

eFigure 2. Calibration and discrimination of the major vascular event (MVE) risk score.

eFigure 3. Hazard ratio for major bleed associated with fifths of major bleed risk score derived from HES admission data and standard factors.

eFigure 4. Observed major vascular event and major bleed (broader definition) rates and their ratios by predicted major vascular event and major bleed risks, after correction to no antiplatelet use.

eFigure 5. Hazard ratios for major bleed and major vascular events associated with fifths of QBleed risk score.

eFigure 6. Observed major vascular event and major bleed rates and their ratios by predicted major vascular event and QBleed risks, after correction to no antiplatelet use.

Data Tables (available in the separate 'Supplementary Data.xlsx' document)

Data Table 1. ICD-10 and OPCS-4 code ranges used to define prior atherosclerotic cardiovascular diseases and incident major vascular events.

Data Table 2. Number of participants with a hospital spell including ICD-10/OPCS-4 code for major bleed.

Data Table 3. Hospital admission and procedures risk factors considered for major vascular event risk: number with factors.

Data Table 4. Hospital admission and procedures risk factors considered for major bleed risk: number with factors.

Data Table 5. Standard and additional risk factors (other than hospital admission and procedures) considered for major bleed risk.

Data Table 6. Risk factors for major vascular events and their parameter estimates for forming the risk score.

Data Table 7. Risk factors for major bleed conditional on major vascular event risk factors and their parameter estimates for forming the risk score.

Data Table 8. Risk factors for major bleed derived from HADP data and standard factors conditional on major vascular event risk factors and their parameter estimates for forming the risk score.

Hammami I, *et al. Heart* 2025;0:1–6. doi: 10.1136/heartjnl-2024-324841

Supplementary methods

eMethods 1: Data preparation

Electronic health records and linkage

The Hospital Episodes Statistics dataset is a repository of data hosted by NHS Digital that relates to in-patient care provided in England. Data submitted on a regular basis by NHS hospital trusts are consolidated, validated and cleaned to produce the Hospital Episode Statistics dataset. The Hospital Episode Statistics data; and the equivalent in Wales, the Patient Episode Database for Wales; and in Scotland, the Scottish Morbidity Records; will jointly be referred to as Hospital Admission Diagnoses or Procedures (HADP). In the HADP dataset, each record contains data relating to a continuous period of care under one consultant known as a Finished Consultant Episode. Each Finished Consultant Episode contains International Classification of Diseases 10th revision (ICD-10) codes and Office of Population Censuses Surveys Classification of Surgical Operations and Procedures 4th revision (OPCS-4) codes along with dates for each procedure. One ICD-10 code is recorded to indicate the primary diagnosis (with up to 20 secondary diagnoses and procedures).

Participants have been linked to HADP and death data, available from about 12 years before recruitment until 30/9/2021 in England (Hospital Episode Statistics), 28/2/2018 in Wales (Patient Episode Database Wales) and 31/7/2021 in Scotland (Scottish Morbidity Record One), providing about 12 years average follow-up after.

Hammami I, *et al. Heart* 2025;0:1–6. doi: 10.1136/heartjnl-2024-324841

Outcomes

For this analysis, Finished Consultant Episodes are combined to form spells of a continuous period of in-patient care provided by a single hospital(1, 2) (WHO 2004, NHS digital 2017). ICD-10 hospital admission diagnoses (recorded in the primary or any secondary position) were used to define first incident major vascular and major bleed events during follow-up and to define prior disease risk factors prior to each year of follow-up.

Outcomes: major vascular event

Major vascular event (MVE) was defined as non-fatal myocardial infarction, non-fatal stroke or transient ischaemic attack, arterial revascularization or vascular death, excluding cerebral haemorrhage (Data Table 1).

Outcomes: major bleed

ICD-10 codes for major bleeds were identified based on ICD-10 code lists used in previous reports to identify bleeding events(3-5) and a review of all ICD-10 codes for any additional relevant bleed codes (Data Table 2). Major bleeds of six types were considered: upper gastrointestinal (GI), lower GI, intracranial, respiratory (including epistaxis and haemoptysis), haematuria (diagnostic code in primary position only) and not elsewhere classified. For hospital admissions for unspecified GI bleeds, GI procedures during the same spell were used to assign the bleeds as upper or lower GI: an unspecified GI bleed in the first diagnostic position together with an upper GI bleed procedure code (G16, G42, G43, G44, G45, G55, G65, G79 and G80) in any position within the same spell was taken as an upper GI bleed, otherwise the unspecified GI bleed was taken as a lower GI bleed. Bleeding sites not used to identify major or minor bleed included: those related to physiological causes

Hammami I, *et al. Heart* 2025;0:1–6. doi: 10.1136/heartjnl-2024-324841

(gynecologic) or rare (bleeding into a joint [haemarthrosis] and bleeding into the pericardium [hemopericardium] or peritoneum [hemoperitoneum]); bleeds defined by the 4th ICD-10 code digit (which is not available in UK Biobank), such as diverticular disease of intestine with bleeding (K57. 31) or intra-medullary bleed (G85.19); hyperplastic polyps and haemorrhoids. Serious/sight-threatening intra-ocular bleeds could not be included as they cannot be identified well from the available hospital admission data because intra-ocular bleeds may not result in an admission and the admission data does not record the severity even when they are captured. Information on transfusions from HADP data cannot be used to determine the seriousness of a bleed because the coding advice is to record transfusions only when they are the sole reason for admission.(6)

First major bleeds following recruitment were defined as intracranial and gastrointestinal bleeds with a duration of hospital spell ≥ 2 days or in death records as the underlying cause. Bleeds were regarded as fatal if a bleed diagnosis was recorded on the death certificate (as the underlying cause of death). A broader definition of major bleed including intracranial bleed and upper GI, lower GI, respiratory, haematuria and site unknown with hospital spell ≥ 1 day was also considered.

Exposures: baseline factors

The 87 baseline factors considered for risk of major bleed are shown in Data Table 5 and include 40 factors classed as 'standard' on the basis that they would be readily available for patients being assessed for cardiovascular risk. For lung function, 'best'

measures for forced expiratory volume in 1 second (FEV1) and forced vital capacity (FVC), defined within UK Biobank were used where available (~70% of participants) and where not available the means of up to 3 readings classed as acceptable were used (as has been previously recommended(7)), reducing the missingness in FEV1 and FVC to about 9%. Alcohol consumption was quantified as the number of UK units of alcohol consumed per week, computed by summing the average weekly intake of each alcohol type. Alcohol consumptions ≥ 120 units per week were set to missing. Biochemistry data that were missing because values were below or above the reportable range were set to the respective lower/upper limit of the reportable range. For urinary microalbumin, serum oestradiol and rheumatoid factor, where a high proportion of values were ‘naturally low’ (i.e. below the laboratory cut-off) rather than ‘missing’, the variables were dichotomized into ‘naturally low’ versus ‘high’. Participants with no haematology data, or no biochemistry data or with any established MVE risk factors (attained age, sex, systolic and diastolic blood pressure, use of blood pressure-lowering medication, body mass index, smoking status, diabetes and non-HDL cholesterol) missing (8.4%) were excluded. Other missing factors were imputed based on random forests (using the MissForest package in R(8)) separately for haematological factors, blood biochemistries, spirometry factors and adiposity markers, within sex strata (eFigure 1), as this approach has previously been found to perform well, particularly on laboratory data.(9) Missing values for hand grip strength, heel bone density T-score, physical activity, alcohol units/week and cognitive factors (3%) were imputed using the mean within 5-year age group and sex strata.

Attained age was categorized into 7-year age-at-risk groups (<50, 50<55; 55<60; 60<65; 65<70; 70<75; ≥75). Measures of lung function were divided by height.

Adiposity markers, systolic and diastolic blood pressure, handgrip strength and heel bone density T-score were expressed in sex-specific standard deviation units and all other continuous measures were expressed in overall standard deviation units (Data Table 5).

Exposure: disease history from electronic health records

A review of all ICD-10 codes not included in the definition of MVE (excluding codes beginning O-Q, U-Z) classified codes as potential vascular risk factors, with the remainder being regarded as potential non-vascular risk factors. ICD-10-based vascular and non-vascular risk factors were grouped into a few well-established conditions(10), and then the remainder grouped by section ranges (Data Tables 3 and 4). Similarly, a review of OPCS-4 codes classified codes as potential vascular risk factors (codes beginning K, L) and non-vascular factors (codes beginning G-J, M). OPCS-4 codes were grouped into categories (Data Tables 3 and 4).

Prior diseases groupings for which <100 participants had a hospitalisation were not considered further (shown in grey in Data Tables 3 and 4). For each year of follow-up, only admissions occurring prior to 28 days before the start of the year were considered (to mitigate against including as prior disease, admissions that were part of closely connected spells to outcomes during the following year). Prior diseases considered for selection with number of participants ≥200 were further categorized by whether they had occurred recently (within 2 years prior to the start of the year of follow-up) or longer ago. Diagnoses that were part of the major or minor bleed definition, with or without an overnight stay were included as risk factors if they occurred before recruitment for major bleeds and up to censoring for minor bleeds.

Hammami I, *et al. Heart* 2025;0:1–6. doi: 10.1136/heartjnl-2024-324841

Diabetes, hypertensive diseases and major vascular events at recruitment were defined using self-reported medical history and HADP information prior to recruitment. An ICD-10-hospital-admission based frailty risk score (excluding ICD-10 codes related to cardiovascular conditions or bleeding (F01, G45, I63, I67, I69, K92 and R31)) was included as an indicator of generalized frailty/comorbidity.(11)

eMethods 2. Identifying vascular predictors of major vascular event and non-vascular predictors of major bleed

Two stages were involved in making the MVE risk score and the non-vascular risk score for major bleed:

1. A 3-step selection process using Cox regression identified risk factors contributing importantly.
2. Using a training/test data approach (internal cross-validation), an absolute risk score for MVE, and a relative risk score for major bleed, were made for each participant.

Stage 1: Outline of the selection algorithm

The sets of variables available for selection are shown in Data Tables 3 for MVE risk and in Data Tables 4 and 5 for major bleed risk. Attained age and the HADP derived factors were updated annually; for other variables only baseline values were available and were used in all years. A three-step selection algorithm which combined computational practicality, statistical significance and the Akaike

information criterion(12) (AIC) was used to sequentially select explanatory variables into a Cox proportional hazards model. The UK Biobank is a very large study with the power to detect many predictors, including many that might contribute relatively little information. Therefore, we sought to implement strict inclusion criteria to avoid the inclusion of predictors that contributed little information. The selection approach was designed to limit the selection of factors to those that added importantly compared to base strong factors of age and sex for major vascular events and the frailty score for major bleed. Selection was based on p-values ($p < 10^{-7}$ for MVE and $p < 10^{-9}$ for major bleed) as this was the criterion available in the stepwise selection in the SAS procedure PROC PHREG, but the p-values were chosen so that a factor on 1 degree of freedom (df) considered for selection would be entered into the model only if it contributed an improvement in AIC $(\Delta\mathrm{AIC})$ of at least 2% of the $\Delta\mathrm{AIC}$ of attained age and sex for MVE risk and 2% of the $\Delta\mathrm{AIC}$ of the frailty risk score for major bleed risk, based on the relationship(13):

$$\Delta\mathrm{AIC} = \mathrm{F}^{-1}_{\chi^2_1}(1-\mathrm{p}) - 2 \quad \text{(Equation 1)}$$

where $\mathrm{F}^{-1}_{\chi^2_1}$ is the inverse cumulative distribution function of a chi-square random variable with $1\ df, \mathrm{p\ is\ the\ p-value}$ and $\mathrm{F}^{-1}_{\chi^2_1}(1-p)$ is the quantile of the χ^2_1 distribution. If $\Delta\mathrm{AIC}_{\mathrm{Comp}}$ denotes the $\Delta\mathrm{AIC}$ of the base predictors then it follows from equation (1) that for a variable (with $1\ \mathrm{df}$) to be entered into the model, it must be significant at the level $\alpha\Delta\mathrm{AIC}$ given by

$$\alpha\Delta\mathrm{AIC} = \mathrm{P}\left(\chi^2_1 > 2 + 0.02\ \Delta\mathrm{AIC}_{\mathrm{Comp}}\right) \quad \text{(Equation 2)}$$

For example, for selecting the major bleed risk factors, the ΔAIC for the frailty score was about 1172. Using equation (2), this corresponded to a significance level

Hammami I, *et al. Heart* 2025;0:1–6. doi: 10.1136/heartjnl-2024-324841

$\alpha\Delta\text{AIC} \approx 10^{-9}$ (based on the accuracy of the survival function [SDF in SAS] used to calculate the small tail areas of the chi-squared distribution).

For computational practicality, this over-arching aim for MVE and for each type of major bleed was implemented in 3 steps as follows:

Step 1: Pre-screening of variables considered for selection individually; variable retained if $\Delta\text{AIC} > 2\% \text{ of } \Delta\text{AIC}_{\text{Comp}}$.

Step 2: Stepwise selection with entry of variables with p-value less than the computed αΔAIC for the type of bleed. The p-value for removal of variables was set to a fixed value of $P>10^{-4}$.

Step 3: Variables that failed the selection criterion in Step 1 were re-evaluated for significance in a stepwise approach similar to Step 2, after including all variables selected in Step 2.

Stage 2: Making the final risk score

Major vascular event risk

As it is the absolute MVE risk that is important when considering prioritization of aspirin treatment, risk scores for the absolute annual MVE risk were formed by fitting Poisson regressions to the established and additionally selected risk factors using a training/test data approach.

To avoid over-fitting from model fitting and evaluation in the same dataset, an internal 10-fold cross-validation was performed. Data were divided into 10 random subsets of equal size. For participants in each subset the log event rate ratios

(betas) for all factors included in the model were estimated using the other 90% of participants.

Analyses were stratified by year of follow-up ($j = 1, \ldots, 12$). For participant i during the follow-up year j, let $Y_{ij} = 1$ or 0 denote the occurrence or otherwise of the outcome under consideration, and let T_{ij} denote the proportion of a year of follow-up the participant is at risk in the stratum during the year j $(0 < T_{ij} \leq 1)$. The logarithm of the expected annual event rate was modelled through the following Poisson regression model:

$$\ln\left(\frac{E(Y_{ij})}{T_{ij}}\right) = \alpha_j + \sum_k \beta_k x_{ijk} \quad \text{(Equation 3)}$$

where $\mathrm{X}_1, \mathrm{X}_2, \ldots, \mathrm{X}_\mathrm{k}$ are the selected risk factors (including any variable that was forced into the model), α_j is the baseline (log) event rate during the period j and x_{ijk} is the value of the kth risk factor (X_k) for participant i during the period j. The log event rate ratios in Equation 3 were used in conjunction with the estimated baseline (log) event rates to predict the average yearly MVE risk P_{ij} for a participant i during the period j, where $P_{ij} = e^{\gamma_{ij}}$ and $\gamma_{ij} = \hat{\alpha}_j + \sum_k \hat{\beta}_k x_{ijk}$. The risk score calculations were run separately among participants with and without known prior atherosclerotic cardiovascular disease. On the basis of P_{ij} for the MVE model, person-years with no known prior atherosclerotic cardiovascular disease were categorised into four groups of predicted risk of MVE <0.2, ≥0.2<0.5, ≥0.5<1 and ≥1 %/y; and those with prior atherosclerotic cardiovascular disease were categorised into two groups <2 and ≥2 %/y.

Hammami I, *et al. Heart* 2025;0:1–6. doi: 10.1136/heartjnl-2024-324841

Assessing calibration and discrimination of the MVE risk score

Calibration of the MVE risk score was assessed by plotting the observed MVE rate (%/y) on the y-axis within deciles of the cross-validated predicted MVE risk score (%/y) on the x-axis.

Discrimination of the MVE risk score was assessed by calculating the c-statistic (as the area under the curve (AUC))(14) based on the predicted probabilities obtained from the Poisson regression model. Logistic regression was applied to these predicted probabilities to calculate the Receiver Operating Characteristic (ROC) curve, from which the c-statistic was derived. The c-statistic was calculated separately for each fold, and the mean c-statistic across all folds was reported as the final measure of discrimination.

Major bleed risk

For major bleed, a predictor of the hazard ratio was calculated, since it is the relative risk of major bleed at a given level of MVE risk that enables classification of participants with higher bleed risk versus lower bleed risk after taking into account vascular risk. A risk score for the hazard ratio of each type of major bleed was formed from the selected non-vascular risk factors with their parameter estimates re-fitted with 10-fold cross-validation (and the same adjustments). Analyses were stratified by year of follow-up (as in the previous section). Person-years at risk of a first major bleed during follow-up, were categorized into fifths according to the predicted major bleed risk separately (i) over years with no known prior atherosclerotic cardiovascular disease and (ii) among participants with a prior atherosclerotic cardiovascular disease at recruitment.

eMethods 3. Correction to no antiplatelet use

The risk factor selection analyses included participants on and not on antiplatelets (with a flag for antiplatelet use) because antiplatelet use might be affected by reverse causality. That is, people with high risk of bleed and having had minor bleeds (possibly not recorded) may selectively not be on antiplatelets. However, to allow for the effects of differences in the percentages of participants on antiplatelets in different subgroups the observed rates of MVE and major bleed within subgroups in Figure 3 and eTable 4 were corrected to no antiplatelet use as follows:

Corrected rate = observed rate *(1/(1+p_a*(RR_a-1)), where p_a is the observed proportion taking antiplatelet therapy and RR_a is the relative risk associated with aspirin, taken as 0.89 (95% CI: [0.84,0.94]) for MVE and 1.43 (95% CI: [1.30,1.56]) for major bleed based on a recently published meta-analysis.(15)
Confidence intervals of the rates were obtained on the log scale using least squares means and were then exponentiated.

The asymptotic standard error (σ) of the ratio estimate was obtained using the first-order delta method as follows

$$\sigma_{ratio} \approx \sqrt{\frac{\sigma^2_{MVE}}{\mu_{bleed}{}^2} + \frac{\mu_{MVE}{}^2 * \sigma^2_{bleed}}{\mu_{bleed}{}^4}}$$

where

μ_{MVE} is the mean MVE rate

μ_{bleed} is the mean major bleed rate

$\sigma^2_{MVE}$is the variance of the MVE rate

Hammami I, *et al. Heart* 2025;0:1–6. doi: 10.1136/heartjnl-2024-324841

σ_{bleed}^2 is the variance of the major bleed rate

The standard error of the rate estimate was approximated by $\sigma_{rate} \approx \sigma_{\log(rate)} * rate$

Note that these approximations did not account for uncertainty around the estimates of the proportion of antiplatelet use.

eMethods 4. Implementation of the QBleed score

The QBleed risk score was implemented using the open source code provided on the QBleed web calculator (https://qbleed.org/). The score was calculated separately in males and females based on 18 out the 21 variables used in the final model(3). These were age, body mass index, Townsend score, smoking status, ethnicity, alcohol intake, previous bleed, oesophageal varices, chronic liver disease or pancreatitis, atrial fibrillation, venous thromboembolism, congestive cardiac failure, treated hypertension, cancer, medication use at baseline including antiplatelets, anticoagulants, non-steroidal anti-inflammatory drugs and corticosteroids. Models also included fractional polynomial terms for age and body mass index.
Fractional polynomial terms for women were $(\text{age}/10)^{-1}$ and $(\text{age}/10)^{3}$; $(\text{body mass index}/10)^{-2}$ and $(\text{body mass index}/10)^{-2}$ ln(body mass index). Fractional polynomial terms for men were $(\text{age}/10)^{-1}$ and $(\text{age}/10)^{3}$ ln(age); $(\text{body mass index}/10)^{-2}$ and $(\text{body mass index}/10)^{-2}$ ln(body mass index). The models for men and women also included interactions between the age terms and anticoagulant use and between age and previous bleed.

References

1. Herbert A, Wijlaars L, Zylbersztejn A, Cromwell D, Hardelid P. Data Resource Profile: Hospital Episode Statistics Admitted Patient Care (HES APC). Int J Epidemiol. 2017;46(4):1093-i.
2. Busby J, Purdy S, Hollingworth W. Calculating hospital length of stay using the Hospital Episode Statistics; a comparison of methodologies. BMC Health Serv Res. 2017;17(1):347.
3. Hippisley-Cox J, Coupland C. Predicting risk of upper gastrointestinal bleed and intracranial bleed with anticoagulants: cohort study to derive and validate the QBleed scores. BMJ. 2014;349:g4606.
4. Rikala M, Kastarinen H, Tiittanen P, Huupponen R, Korhonen MJ. Natural history of bleeding and characteristics of early bleeders among warfarin initiators - a cohort study in Finland. Clin Epidemiol. 2016;8:23-35.
5. Selak V, Kerr A, Poppe K, Wu B, Harwood M, Grey C, et al. Annual Risk of Major Bleeding Among Persons Without Cardiovascular Disease Not Receiving Antiplatelet Therapy. JAMA. 2018;319(24):2507-20.
6. Terminology and Classifications Delivery Service. National Clinical Coding Standards OPCS-4: Accurate data for quality information 2021 [Available from: https://classbrowser.nhs.uk/ref_books/OPCS-4.9_NCCS-2021.pdf.
7. Gupta RP, Strachan DP. Ventilatory function as a predictor of mortality in lifelong non-smokers: evidence from large British cohort studies. BMJ Open. 2017;7(7):e015381.
8. Stekhoven DJ, Buhlmann P. MissForest--non-parametric missing value imputation for mixed-type data. Bioinformatics. 2012;28(1):112-8.
9. Waljee AK, Mukherjee A, Singal AG, Zhang Y, Warren J, Balis U, et al. Comparison of imputation methods for missing laboratory data in medicine. BMJ Open. 2013;3(8).
10. Stanley J, Sarfati D. The new measuring multimorbidity index predicted mortality better than Charlson and Elixhauser indices among the general population. J Clin Epidemiol. 2017;92:99-110.
11. Gilbert T, Neuburger J, Kraindler J, Keeble E, Smith P, Ariti C, et al. Development and validation of a Hospital Frailty Risk Score focusing on older people in acute care settings using electronic hospital records: an observational study. Lancet. 2018;391(10132):1775-82.
12. Akaike H. New Look at Statistical-Model Identification. Ieee T Automat Contr. 1974;Ac19(6):716-23.
13. Murtaugh PA. In defense of P values. Ecology. 2014;95(3):611-7.
14. Harrell FE, Jr., Lee KL, Mark DB. Multivariable prognostic models: issues in developing models, evaluating assumptions and adequacy, and measuring and reducing errors. Stat Med. 1996;15(4):361-87.
15. Zheng SL, Roddick AJ. Association of Aspirin Use for Primary Prevention With Cardiovascular Events and Bleeding Events: A Systematic Review and Meta-analysis. JAMA. 2019;321(3):277-87.
16. Score working group and ESC Cardiovascular risk collaboration. SCORE2 risk prediction algorithms: new models to estimate 10-year risk of cardiovascular disease in Europe. Eur Heart J. 2021;42(25):2439-54.

eTable 1. Observed major vascular event rates by predicted major vascular event risk score.

Predicted major vascular event risk, %/y	%	N with event	Rate, %/y (95% CI)
**<0.2**	40.5	1982	0.10 (0.09, 0.10)
**≥0.2<0.5**	35.9	6169	0.34 (0.33, 0.35)
**≥0.5<1**	18.6	6772	0.73 (0.71, 0.75)
**≥1**	5.0	3387	1.38 (1.34, 1.43)

Abbreviation: CI: confidence interval.

Hammami I, *et al. Heart* 2025;0:1–6. doi: 10.1136/heartjnl-2024-324841

eTable 2. Observed major vascular event rates by predicted major vascular event risk scores derived from established and additional vascular factors and SCORE2.

Tenths of risk Score	Major vascular event risk score		Major vascular event risk score based on established vascular risk factors only *		SCORE2**	
	N with event	Rate, %/y (95% CI)	N with event	Rate, %/y (95% CI)	N with event	Rate, %/y (95% CI)
1	195	0.038 (0.033, 0.043)	212	0.041 (0.036, 0.047)	205	0.039 (0.034, 0.045)
2	353	0.069 (0.062, 0.076)	360	0.070 (0.063, 0.078)	366	0.071 (0.064, 0.079)
3	585	0.114 (0.105, 0.124)	622	0.121 (0.112, 0.131)	612	0.120 (0.110, 0.129)
4	836	0.164 (0.153, 0.175)	872	0.171 (0.160, 0.182)	852	0.167 (0.156, 0.179)
5	1161	0.228 (0.216, 0.242)	1196	0.235 (0.222, 0.249)	1204	0.237 (0.224, 0.251)
6	1498	0.296 (0.281, 0.312)	1533	0.303 (0.288, 0.319)	1583	0.312 (0.297, 0.328)
7	2078	0.413 (0.395, 0.431)	2087	0.415 (0.397, 0.433)	1982	0.394 (0.377, 0.411)
8	2560	0.513 (0.493, 0.533)	2554	0.512 (0.492, 0.532)	2678	0.536 (0.516, 0.557)
9	3545	0.718 (0.695, 0.742)	3599	0.730 (0.706, 0.754)	3561	0.721 (0.698, 0.745)
10	5499	1.139 (1.109, 1.170)	5275	1.091 (1.062, 1.121)	5267	1.098 (1.068, 1.128)

Abbreviation: CI: confidence interval.
*Based on established risk factors: age, sex, systolic and diastolic blood pressure, use of blood pressure lowering medications, body mass index, smoking status, diabetes status, and non-HDL cholesterol.
**Based on sex-specific models, including age, smoking status, systolic blood pressure, total- and HDL-cholesterol. diabetes status, and age interactions.(16)

Hammami I, *et al. Heart* 2025;0:1–6. doi: 10.1136/heartjnl-2024-324841

eTable 3. Hazard ratios for lower gastrointestinal (GI) bleed, broader major bleed, and major vascular events, respectively, associated with fifths of non-vascular risk score for major bleed. Hazard ratios for major bleeds are adjusted for attained age, sex, anticoagulation and antiplatelet use and major vascular event risk score (both continuous and categorical forms). Hazard ratios for major vascular event are adjusted for attained age, sex, anticoagulation and antiplatelet use. Abbreviation: CI: confidence interval.

Fifths of non-vascular risk score for major bleed	Lower GI bleed		Broader major bleed		Major vascular event	
	N with event	Hazard ratio (95% CI)	N with event	Hazard ratio (95% CI)	N with event	Hazard ratio (95% CI)
1	98	1.00 (0.82, 1.23)	592	1.00 (0.92, 1.09)	2316	1.00 (0.96, 1.04)
2	99	0.95 (0.78, 1.16)	695	1.11 (1.03, 1.19)	2842	1.11 (1.07, 1.15)
3	123	1.13 (0.95, 1.35)	1036	1.58 (1.48, 1.68)	3141	1.15 (1.11, 1.19)
4	192	1.70 (1.48, 1.96)	1517	2.21 (2.11, 2.33)	3983	1.36 (1.32, 1.41)
5	652	5.11 (4.70, 5.56)	5426	7.08 (6.88, 7.29)	6028	1.88 (1.83, 1.93)

Hammami I, *et al. Heart* 2025;0:1–6. doi: 10.1136/heartjnl-2024-324841

eTable 4. Observed major vascular event and major bleed rates by predicted major vascular event and major bleed risks after correction to no antiplatelet use. Abbreviation: CI: confidence interval; MVE: major vascular event.

		Fifths of predicted major bleed risk					
		1		2-4		5	
**Predicted major vascular event risk, %/y**	**Event**	**N with event**	**Rate, %/y (95% CI)**	**N with event**	**Rate, %/y (95% CI)**	**N with event**	**Rate, %/y (95% CI)**
**<0.5**	MVE	1204	0.16 (0.15, 0.17)	4446	0.19 (0.19, 0.20)	2446	0.32 (0.31, 0.34)
	Major Bleed	224	0.03 (0.02, 0.03)	1046	0.04 (0.04, 0.05)	1281	0.16 (0.15, 0.17)
	Ratio		5.53 (4.74, 6.32)		4.40 (4.11, 4.70)		2.00 (1.86, 2.13)
**≥0.5<1**	MVE	1139	0.63 (0.59, 0.67)	3775	0.70 (0.67, 0.72)	1766	1.01 (0.96, 1.06)
	Major Bleed	176	0.09 (0.08, 0.10)	728	0.12 (0.12, 0.13)	755	0.39 (0.37, 0.42)
	Ratio		6.97 (5.87, 8.08)		5.63 (5.18, 6.07)		2.55 (2.34, 2.77)
**≥1**	MVE	569	1.21 (1.11, 1.31)	1899	1.36 (1.30, 1.42)	831	1.88 (1.76, 2.01)
	Major Bleed	128	0.24 (0.21, 0.29)	423	0.27 (0.24, 0.30)	451	0.90 (0.82, 0.98)
	Ratio		4.95 (4.00, 5.90)		5.04 (4.51, 5.58)		2.09 (1.85, 2.33)

Hammami I, *et al. Heart* 2025;0:1–6. doi: 10.1136/heartjnl-2024-324841

eTable 5. Proportion of proton pump inhibitors use (%) at baseline by predicted major vascular event and major bleed risks.

Predicted major vascular event risk, %/y	Fifths of predicted major bleed risk		
	**1**	**2-4**	**5**
**<0.5**	4.4	7.5	17.0
**≥0.5<1**	7.0	10.5	20.2
**≥1**	7.9	11.1	20.9

Hammami I, *et al. Heart* 2025;0:1–6. doi: 10.1136/heartjnl-2024-324841

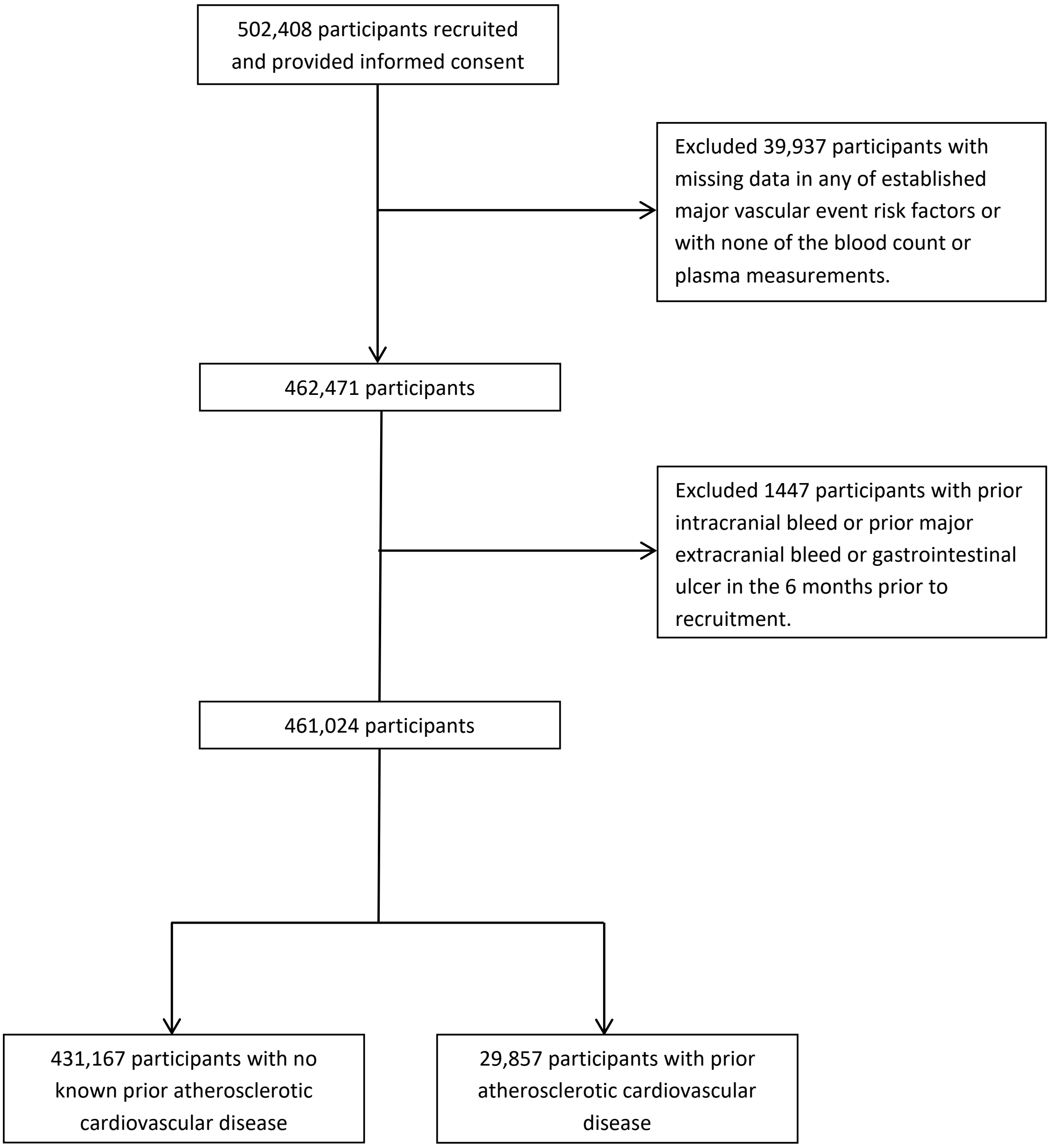

eFigure 1. Flow diagram for participants included and the various reasons for exclusion.

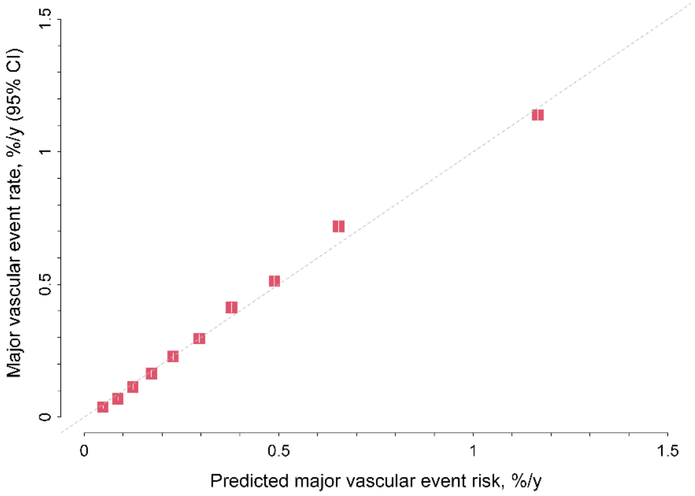

Fold	c-statistic
1	0.770
2	0.759
3	0.787
4	0.210
5	0.773
6	0.778
7	0.779
8	0.799
9	0.791
10	0.810
Mean	0.726

The cross-validated MVE risk score demonstrated good calibration, with the predicted and observed rates aligning closely along a 45-degree line. The mean c-statistic for the MVE risk score across the 10-fold cross-validation was 0.726, indicating a good level of predictive performance.

eFigure 2. Calibration and discrimination of the major vascular event (MVE) risk score.

Hammami I, *et al. Heart* 2025;0:1–6. doi: 10.1136/heartjnl-2024-324841

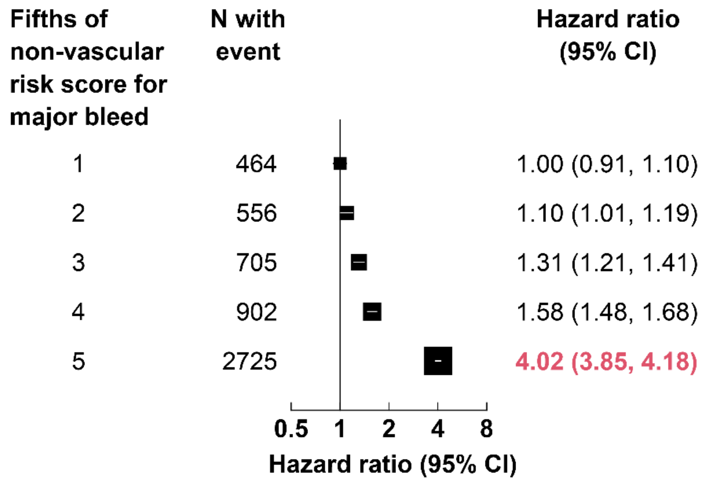

eFigure 3. Hazard ratio for major bleed associated with fifths of major bleed risk score derived from HES admission data and standard factors. Hazard ratios are adjusted for attained age, sex, anticoagulation and antiplatelet use and major vascular event risk score (both continuous and categorical forms). Abbreviation: CI: confidence interval.

Hammami I, *et al. Heart* 2025;0:1–6. doi: 10.1136/heartjnl-2024-324841

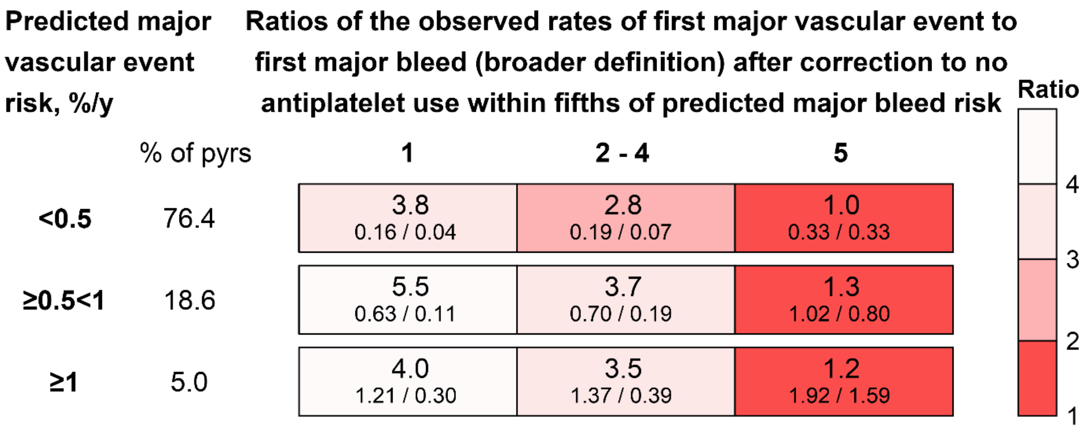

		Fifths of predicted major bleed risk					
		1		2-4		5	
Predicted major vascular event risk, %/y	Event	N with event	Rate, %/y (95% CI)	N with event	Rate, %/y (95% CI)	N with event	Rate, %/y (95% CI)
**<0.5**	MVE	1204	0.16 (0.15, 0.17)	4440	0.19 (0.19, 0.20)	2452	0.33 (0.31, 0.34)
	Major Bleed	327	0.04 (0.04, 0.05)	1649	0.07 (0.07, 0.07)	2599	0.33 (0.32, 0.34)
	Ratio		3.79 (3.33, 4.25)		2.79 (2.63, 2.95)		0.98 (0.93, 1.04)
**≥0.5<1**	MVE	1131	0.63 (0.59, 0.67)	3769	0.70 (0.68, 0.72)	1780	1.02 (0.98, 1.07)
	Major Bleed	222	0.11 (0.10, 0.13)	1114	0.19 (0.18, 0.20)	1523	0.80 (0.76, 0.84)
	Ratio		5.49 (4.70, 6.28)		3.67 (3.42, 3.91)		1.27 (1.19, 1.36)
**≥1**	MVE	563	1.21 (1.11, 1.31)	1894	1.37 (1.31, 1.43)	842	1.92 (1.79, 2.05)
	Major Bleed	155	0.30 (0.26, 0.35)	600	0.39 (0.36, 0.42)	788	1.59 (1.48, 1.71)
	Ratio		4.05 (3.33, 4.77)		3.54 (3.22, 3.87)		1.21 (1.09, 1.32)

eFigure 4. Observed major vascular event and major bleed (broader definition) rates and their ratios by predicted major vascular event and major bleed risks, after correction to no antiplatelet use. Predicted major bleed risk cutoffs were based on major bleed risk score quintiles calculated separately within each major vascular event risk group. Values in bars are the ratio (in large type) and the major vascular event rate (%/y) / major bleed rate (%/y) underneath. Abbreviation: pyrs: person-years at risk.

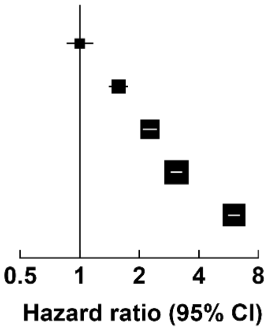

Fifths of QBleed risk score for major bleed	Major bleed: N with event	Major bleed: Hazard ratio (95% CI)	Major vascular event: N with event	Major vascular event: Hazard ratio (95% CI)
1	329	1.00 (0.86, 1.16)	906	1.00 (0.92, 1.09)
2	490	1.57 (1.41, 1.74)	1847	1.59 (1.50, 1.67)
3	746	2.26 (2.10, 2.43)	2975	2.25 (2.17, 2.34)
4	1124	3.08 (2.91, 3.26)	4584	2.97 (2.89, 3.05)
5	2663	**6.02 (5.66, 6.40)**	7998	**4.24 (4.10, 4.38)**

eFigure 5. Hazard ratios for major bleed and major vascular events associated with fifths of QBleed risk score. Hazard ratios for major bleeds are adjusted for attained age, sex, anticoagulation and antiplatelet use and major vascular event risk score (both continuous and categorical forms). Hazard ratios for major vascular event are adjusted for attained age, sex, anticoagulation and antiplatelet use. Hazard ratios are plotted as squares, with the size of each square proportional to the amount of statistical information that was available; the horizontal lines represent 95% confidence intervals (CI) based on floating absolute risks. The CIs for the top fifth relative to the bottom fifth as a fixed reference group (without floating absolute risks) are 6.02 (5.09, 7.12) for major bleed and 4.24 (3.86, 4.66) for major vascular event.

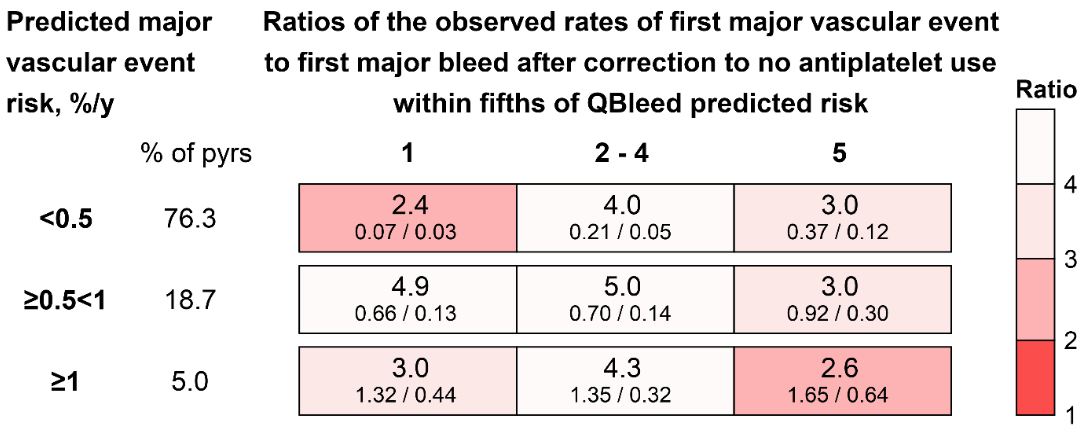

		Fifths of QBleed predicted risk					
		1		2-4		5	
**Predicted major vascular event risk, %/y**	**Event**	**N with event**	**Rate, %/y (95% CI)**	**N with event**	**Rate, %/y (95% CI)**	**N with event**	**Rate, %/y (95% CI)**
**<0.5**	MVE	560	0.07 (0.07, 0.08)	4747	0.21 (0.20, 0.21)	2789	0.37 (0.36, 0.39)
	Major Bleed	234	0.03 (0.03, 0.03)	1232	0.05 (0.05, 0.06)	1080	0.12 (0.12, 0.13)
	Ratio		2.40 (2.03, 2.77)		3.97 (3.73, 4.22)		3.02 (2.81, 3.23)
**≥0.5<1**	MVE	1162	0.66 (0.63, 0.70)	3873	0.70 (0.68, 0.72)	1645	0.92 (0.87, 0.96)
	Major Bleed	237	0.13 (0.12, 0.15)	796	0.14 (0.13, 0.15)	624	0.30 (0.28, 0.33)
	Ratio		4.95 (4.26, 5.64)		4.96 (4.59, 5.34)		3.04 (2.76, 3.32)
**≥1**	MVE	605	1.32 (1.22, 1.43)	1938	1.35 (1.29, 1.41)	756	1.65 (1.54, 1.77)
	Major Bleed	202	0.44 (0.38, 0.50)	461	0.32 (0.29, 0.35)	335	0.64 (0.57, 0.71)
	Ratio		3.02 (2.54, 3.51)		4.28 (3.84, 4.71)		2.59 (2.26, 2.92)

eFigure 6. Observed major vascular event and major bleed rates and their ratios by predicted major vascular event and QBleed risks, after correction to no antiplatelet use, in participants with no known prior atherosclerotic cardiovascular diseases. Predicted QBleed risk cutoffs were based on QBleed risk score quintiles calculated separately within each major vascular event risk group. Values in bars are the ratio (in large type) and the major vascular event rate (%/y) / major bleed rate (%/y) underneath. Abbreviation: pyrs: person-years at risk.
